# Iron and sulphur regulate carbon dioxide emissions in drained coastal peatlands of The Netherlands

**DOI:** 10.1007/s10533-025-01303-x

**Published:** 2026-01-09

**Authors:** Duygu Tolunay, Gizem Ergut, Levi Simon, Gilles Erkens, George A. Kowalchuk, Mariet M. Hefting

**Affiliations:** 1 Ecology and Biodiversity, Institute of Environmental Biology, Utrecht University, Padualaan 8, 3584 CH Utrecht, The Netherlands; 2 Deltares Research Institute, P.O. Box 85467, 3508 AL Utrecht, The Netherlands; 3 Amsterdam Institute for Life and Environment (A-LIFE), Systems Ecology Section, Vrije Universiteit Amsterdam, Van der Boechorstraat 3, 1081 BT Amsterdam, The Netherlands

**Keywords:** Peatlands, Redox, Carbon, Iron, Sulphur, Decomposition

## Abstract

Fluctuating groundwater levels in drained peatlands create a transition zone with seasonally changing oxygen availability. This zone drives dynamic iron (Fe) and sulphur (S) cycling under alternating anoxic and oxic conditions, influencing decomposition rates. This study investigated how Fe and S affect decomposition rates and resulting carbon dioxide (CO_2_) emissions under fluctuating redox conditions in transition zone. In a controlled laboratory experiment, peat samples from two drained Dutch coastal peatlands were amended with ferric iron (Fe^3+^) and sulphate (SO_4_^2−^) and incubated anoxically to mimic high groundwater tables. This was followed by an oxic phase simulating groundwater table drops. The cycle was repeated with lactate addition to replenish labile carbon. Carbon dioxide emission rates were monitored continuously throughout the anoxic–oxic cycles. Water soluble Fe and S concentrations, exoenzyme activities, and pH were measured before and after the experiment. Carbon dioxide emission rates increased under anoxic conditions with Fe^3+^ and SO_4_^2−^ amendments potentially due to stimulation of microbial activity using these compounds as alternative electron acceptors. Short-term oxygenation suppressed emissions compared to controls without amendments. Water-soluble Fe remained stable across treatments, while water-soluble S concentrations changed significantly from initial levels. Exoenzyme activities were primarily influenced by pH, with minimal effects from amendments. The findings show that transition zone is an active redox zone where decomposition dynamics are determined by available electron acceptors in the system, influencing greenhouse gas (GHG) emissions from managed peatlands. This zone should be integrated into future models to improve the accuracy of reporting national GHG emissions.

**Supplementary Information:**

The online version contains supplementary material available at 10.1007/s10533-025-01303-x.

## Introduction

Drained coastal peatlands in the Netherlands are dynamic ecosystems influenced by groundwater table fluctuations caused by water management and increased evaporation due to long lasting summer droughts with climate change (Aben et al. 2024; Erkens et al. 2016; Oude Essink et al. 2010; van Diggelen et al. 2020). Fluctuating groundwater tables create a dynamic layer in the peat profile which is often referred to as the transition zone, where the peat soil alternates between saturated and unsaturated conditions. This zone, typically ranging from 30 cm to a metre in thickness, is a hotspot for biogeochemical processes, particularly those involving iron (Fe) and sulphur (S), (Harpenslager et al. 2024; Zak et al. 2010). Coastal peatlands in the Netherlands are rich in Fe and S, which can be transported from deeper layers to the transition zone during high groundwater table periods (Koebsch et al. 2019; Vermaat et al. 2016). In drained coastal peatlands, iron typically enters the ecosystem through seepage from deep groundwater and surface runoff. Iron can exist in various forms such as soluble ferrous iron (Fe^2+^), less soluble ferric iron (Fe^3+^), and complexes formed with organic matter, phosphorus and sulphur compounds. The speciation and mobility of iron in these environments are influenced by redox conditions and interactions with organic matter (Bhattacharyya et al. 2018; Huang et al. 2021; Johnson et al. 2012; Ward and Crichton 2015). Sulphur, on the other hand, reaches peatland ecosystems via seawater intrusion, seepage water and atmospheric deposition, and is mainly present in the form of sulphate (SO_4_^2−^) and organic sulphur compounds (Chen et al. 2020; Li et al. 2012; Negassa et al. 2022; Yu et al. 2016).

Under anoxic conditions, ferric iron (Fe^3+^) can be reduced to ferrous iron (Fe^2+^) through microbial activity, with iron-reducing bacteria (IRB) using Fe^3+^ as an alternative terminal electron acceptor (ATEA, Table 1). The reduction of Fe^3+^ is accompanied by carbon oxidation, resulting in carbon dioxide (CO_2_) emissions (Melton et al. 2014; Xu et al. 2021). Apart from microbial processes, autochemical reactions, such as Fenton chemistry, can occur, facilitating spontaneous redox reactions involving iron. These abiotic reactions produce oxidants which can attack persistent organic compounds (such as phenolics) and further promote organic matter decomposition (Du et al. 2020; Merino et al. 2020; Yu and Kuzyakov 2021). Under anoxic conditions, sulphate-reducing bacteria (SRB) utilise sulphate (SO_4_^2−^) as an ATEA to degrade organic matter, producing hydrogen sulphide (H_2_S) and CO_2_ as metabolic byproducts. This process contributes significantly to anaerobic carbon mineralization, with studies indicating that dissimilatory sulphate reduction can account for up to 36–50% of this activity in freshwater wetlands (Boothroyd et al. 2021; Lehnert et al. 2024; Pester et al. 2012). The rates of anaerobic processes are slower compared to aerobic decomposition pathways, but they can be altered by increasing the concentrations of ATEAs or changing the redox conditions through drainage (Blodau et al. 2007; Duverger et al. 2020; Lovleyt and Klug 1983; Lowe and Bustin 1985; Vermaat et al. 2016).

**Table 1:** Abiotic and biotic iron and sulphur redox reactions that can take place in drained peatlands (modified fromCole 2013; Fenchel et al. 2012a; Findlay and Strayer 2013)

Reaction	Type	Equation	Description
Iron oxidation	Autochemical (Abiotic)	4 Fe^2+^ + O_2_ + 4H⁺ → 4 Fe^3+^ + 2 H_2_O	Occurs spontaneously upon oxygen exposure
Iron oxidation	Microbial	4 Fe^2+^ + O_2_ + 10H_2_O → 4Fe(OH)_3_ + 8H^+^	By iron-oxidizing bacteria (e.g., *Gallionella*)
Iron reduction	Microbial	Fe(OH)_3_ + 3H^+^ + e^−^ → Fe^2+^ + 3H_2_O	By iron-reducing bacteria (e.g., *Geobacter*)
Sulphide oxidation	Autochemical (Abiotic)	HS^−^ + 2O_2_ → SO_4_^2−^ + H^+^	Occurs spontaneously upon oxygen exposure
Sulphide oxidation	Microbial	HS^−^ + 2O_2_ → SO_4_^2−^ + H^+^	By sulphur-oxidizing bacteria (e.g., *Beggiatoa*)
Sulphate reduction	Microbial	SO_4_^2−^ + 10H^+^ + 8e^−^ → HS^−^ + 4H_2_O	By sulphate-reducing bacteria (e.g., *Desulfovibrio*)

Draining peat changes the redox conditions with the intrusion of oxygen, whereby Fe^2+^ in the system is oxidised to Fe^3+^, either spontaneously or catalysed by microorganisms (Ionescu et al. 2015; Küsel et al. 2008)*.* The *Iron Gate* hypothesis states that Fe^3+^ complexes with lignin-like compounds, reduce phenol oxidase activity and provide protection to organic matter under oxic conditions (Wang et al. 2017). With oxygen intrusion, reduced sulphur species such as H_2_S are oxidised to SO_4_^2−^, facilitated by sulphur-oxidizing bacteria (Fenchel et al. 2012b). While increased sulphate concentrations in the system can lead to eutrophication in adjacent water bodies by interfering with iron phosphorus chemistry and stimulating anaerobic decomposition of organic matter (Smolders et al. 2010), oxidation of ferrous sulphide minerals (e.g. pyrite) can reduce the pH of the system, and potentially limit heterotrophic aerobic microbial activity and slow organic matter decomposition in the short term (Boothroyd et al. 2021; Duan et al. 2023).

After a dry period, when the transition zone again becomes water-saturated, anoxic conditions return, reactivating microbial reduction pathways for both iron and sulphur. Thus, water fluctuations act like a charging battery for iron and sulphur cycles. The “charged battery” under oxic conditions can be utilised under anoxic conditions to continue degrading organic matter. High decomposition rates under anoxic conditions following rewetting have been reported in several studies, supporting this “charging battery” concept (Emsens et al. 2016; Fenner and Freeman 2011; van De Riet et al. 2013). This phenomenon has also been explained by the *Enzymic latch* hypothesis, which suggests that phenolic compounds in peat inhibit extracellular hydrolytic enzymes produced by microorganisms, thereby protecting organic matter from decomposition under anoxic conditions (Freeman et al. 2001). However, oxygen intrusion can activate oxidative exoenzymes that degrade these phenolic compounds, effectively “removing the latch.” When anoxic conditions return, the reduced concentrations of phenolic compounds, along with increased nutrients, pH, and labile carbon resulting from aerobic decomposition during low water table periods, stimulate anaerobic decomposition (Fenner and Freeman 2011).

Rising sea levels, saltwater intrusion, and anthropogenic inputs are increasing the availability of alternative terminal electron acceptors like iron and sulphur in drained coastal peatlands. At the same time, management responses to droughts, such as rewetting with brackish water, can further enhance these inputs. These changes intensify redox fluctuations within the peat profile, particularly in the transition zone between oxic and anoxic layers. This zone is often overlooked in greenhouse gas (GHG) models, which typically associate CO_2_ emissions with oxic depth alone. However, the transition zone is biogeochemically active and may contribute significantly to total CO_2_ fluxes. By quantifying CO_2_ emissions from this zone and assessing the influence of elevated iron and sulphur concentrations, this study addresses a critical gap in our understanding of managed peatland carbon dynamics. It also anticipates future risks linked to sea level rise and salinisation. Conducting a laboratory incubation experiment that mimics redox fluctuations in the transition zone, we test the hypothesis that iron and sulphur enhance CO_2_ production under anoxic conditions while suppressing it during short oxic phase.

## Materials and methods

### Field sampling

Peat samples were collected from two drained coastal peat meadow sites in the Netherlands used for dairy farming. The Assendelft (ASD) site in North Holland (52° 28′ 33.0″ N, 4° 44′ 24.5″ E) is a peatland where the top 0.3 m of clayey peat overlays peat layers that extend to a depth of approximately 2.0 m. The peat beneath the clay-rich layer exhibits a degradation level of H6 to H8, according to the Van Post scale (Von Post 1922), within the 0.3 to 0.5 m depth range, which represents the transition zone. The peat matrix in this area is primarily composed of sedge and reed mixture. The average groundwater level fluctuates between 18.5 and 55 cm below ground level, and the site is subject to upward seepage conditions. Groundwater in this region is slightly brackish and rich in sulphur and iron, with measured average pore-water concentrations from oxic and anoxic depths of total iron (Fe) at approximately 0.2 mmol/L and sulphate (SO_4_^2−^) at 16.8 mmol/L (Boonman et al. 2022; Harpenslager et al. 2024). Further details regarding the Assendelft field site are available in (Boonman 2024; Erkens et al. 2021) (Table 2; Table S1).

**Table 2:** The characteristics of transition zone of ASD and ROU field sites and range of pore water chemistry data measured at a depth 45 cm between 2020 and 2022 (Boonman 2024, Table S1)

Site	Depth (cm)	Peat type	Bulk density (g/cm^3^)	Carbon density (g/g)	LOI (%)	pH	DOC (mmol/L)	S^2−^ (mmol/L)
ASD	30–50	Sedge/reed	0.3	43.3	75	4.1–6.8	2.6–34.0	0.02–17.5
ROU	40–60	Sedge/reed	0.4	32.2	56	5.8–6.6	6.5–26.1	0.004–2.217

The Rouveen (ROU) site (52° 37′ 59.5″ N, 6° 05′ 22.2″ E) has a similar soil depth profile. The uppermost layer, from 5 to 10 cm, contains clayey peat with organic matter content of approximately 30%. Beneath the clay layer is a peat deposit reaching depths of 3.25 to 3.60 m. The transition zone, spanning a depth of 0.4 to 0.6 m, shows degradation levels ranging from H4 to H8 on the Von Post scale. The peat at this site is characterised by sedge, reed and occasionally wood fragments. Unlike the ASD site, a water infiltration system has been installed approximately 65 to 70 cm below the surface, which can also act as a drainage system. ROU is also an upward seepage site. Average pore-water concentrations from oxic and anoxic depths for total Fe and SO_4_^2−^ are approximately 0.2 mmol/L and 0.5 mmol/L, respectively. Further details regarding the ROU field site are available in Boonman (2024), Erkens et al. (2021), Tolunay et al. (2024) (Table 2; Table S1).

Six peat samples, spaced 30 cm apart, were collected as field replicates from 30 to 50 cm depth at the ASD site and 40 to 60 cm at the ROU site using a 6 cm wide soil gouge auger (Eijkelkamp, Breda, The Netherlands) (Table 2). Sampling was conducted under oxic conditions, as the groundwater table was low during the sampling period. The collected samples were immediately transferred to the laboratory in a cooling box and maintained at approximately 4 °C until further analysis.

### Experimental design

**Fig. 1:**
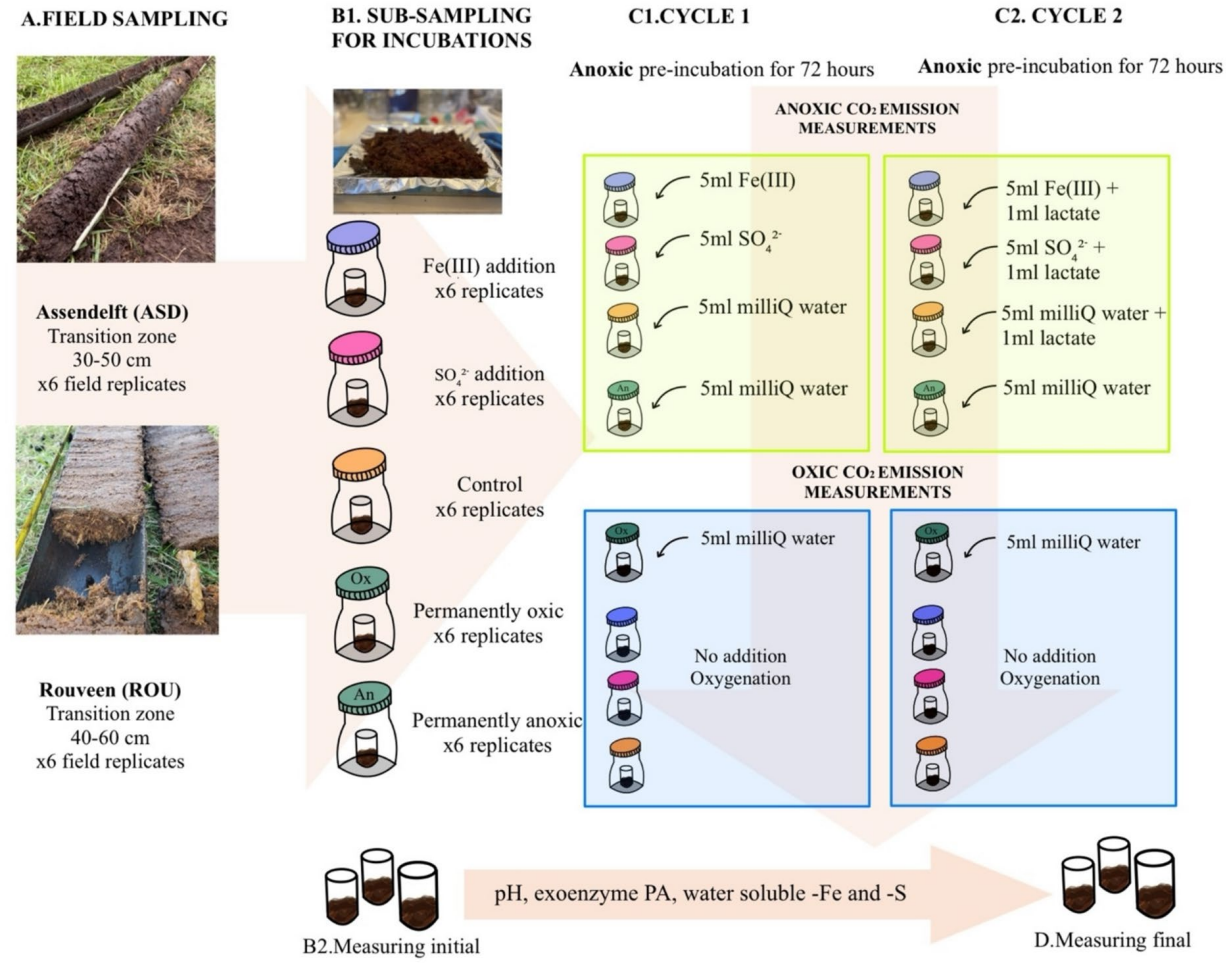
The design and flow of the experiment for the ASD and ROU peat samples: **A** collecting peat samples from the transition zones. **B** Homogenising and subsampling the peat samples in the laboratory. **B1** Measuring the initial/field conditions for several parameters before the incubation experiment. **C1** The first cycle of the incubation experiment consists of pre-incubation under anoxic conditions, followed by anoxic and oxic measurements. **C2** The second cycle follows the same flow as the first cycle, with the addition of lactate as an electron donor, same samples were used for the second cycle. **D** Measurements of parameters are taken at the end of the incubation experiment

In the laboratory, each field replicate was thoroughly mixed individually to ensure its homogeneity under oxic conditions. Subsamples were then taken from each replicate to measure initial field conditions for pH, extracellular enzyme potential activities (exoenzyme PA), and water-soluble iron (Fe) and sulphur (S) concentrations. The remaining peat sample was divided into five sets with six replicates each to evaluate the effects of electron donors (organic matter) and acceptors (O_2_, ferric iron—Fe^3+^ and sulphate—SO_4_^2−^) under oxic and anoxic conditions. Each set consisted of 30 ± 0.1 g of fresh peat, placed in 50 mL glass beakers, which were then transferred into 500 mL Duran bottles with a layer of perlite at the bottom to maintain stable moisture levels during incubations. The experimental conditions included Fe^3+^ amendment, SO_4_^2−^ amendment, and a control group. In addition, two sets of bottles were prepared to investigate the depletion of electron donors and acceptors under permanently oxic (permOx) and permanently anoxic (permAn) conditions (Fig. 1).

The prepared oxic samples except permOx samples were placed in an anoxic chamber (Vinyl Anaerobic Chamber, Coy Laboratory Products, USA) for a 72-h pre-incubation period to create an oxygen-free environment. During the first 8 h, the bottle lids were left open to ensure that the anoxic atmosphere fully permeated the samples and removed any residual oxygen. Following this, the lids were placed loosely on the bottles to allow for limited gas exchange and to decrease water loss. Before initiating the experiment, the lids were opened for 1 h to refresh the anoxic headspace. Throughout the experiment, the temperature was kept stable at 22.5 °C.

The incubation experiment began with the addition of electron acceptors and/or water inside the anoxic chamber. For Fe^3+^ treatment, 5 mL of 50 µM iron chloride (FeCl_3_, Sigma-Aldrich, MW 162.60 g/mol) was added to the respective samples, while 150 µM of magnesium sulphate heptahydrate (MgSO_4_·7H_2_O, Sigma-Aldrich, MW 246.47 g/mol) was used for sulphate treatment. The control samples received 5 mL of Milli-Q water. These concentrations were chosen based on field measurements obtained by other studies (Boonman 2024). After the addition of these solutions, samples were immediately connected to an anoxic respirometer to monitor CO_2_ production in the headspace over time. PermAn samples were incubated in the anoxic chamber and were only connected to the anoxic respirometer along with the other anoxic measurements. In contrast, PermOx samples were kept in an incubator at 22.5 °C and were connected only to the oxic respirometer, together with the oxidised samples, to measure carbon dioxide (CO_2_) emissions. The duration of CO_2_ measurement varied from 5 to 7 days, depending on the point at which stable CO_2_ emissions were reached. Following the anoxic incubation, the samples were oxidised by opening the lids and transferring them to oxic respirometer. Carbon dioxide emissions and O_2_ concentrations (≈ 21%) at the head space were monitored under oxic conditions. This completed the first experimental cycle (Cy1). The same samples were then returned to the anoxic chamber to bring them again to anoxic phase. For the second cycle (Cy2), in addition to the continued Fe^3+^ and SO_4_^2−^ amendments, 1 mL 0.3 M lactate was added as an electron donor to all samples, except those in the permAn or permOx conditions. The measurement sequence during the Cy2 was same to that of the Cy1.

At the end of the experiment, all samples were analysed for pH, exoenzyme PA, carbon content, and concentrations of water-soluble iron and water-soluble sulphur. These measurements were compared to the initial field conditions to assess changes resulting from the experimental treatments.

### Measured parameters

#### Carbon dioxide emission rates (µg C-CO_2_/g C/h)

The carbon dioxide (CO_2_) emission rates for both aerobic and anaerobic activity were measured by monitoring CO_2_ accumulation in the headspace using a respirometer (Biometric Systems, Germany; Biont Research, The Netherlands). The 500 mL bottles containing the incubated samples were connected to the respirometer, and CO_2_ emissions were recorded at intervals of up to 120 min. The system automatically flushed the headspace with nitrogen gas for anoxic measurements or air for oxic measurements when CO_2_ levels reached 0.4 ppm. After Cy2, 10 g of fresh samples were oven-dried at 70 °C for 72 h to determine their dry weight and check the moisture content in the samples. 0.5 mg of the dried samples was later used to measure the carbon content of the samples using an elemental analyser (FlashSmart™ Elemental Analyser, Thermo Fisher Scientific, Inc., USA). The CO_2_ emission rates are expressed in units of µg C-CO_2_/g C/h.

#### Concentrations of water-soluble iron and sulphur (ppmv)

Water soluble iron (Fe) and sulphur (S) concentrations in peat samples were measured using a Thermo Scientific iCAP 6000 Series ICP Emission Spectrometer. Five grams of fresh peat sample, before and after the incubation experiment, were weighed and mixed with 30 mL of Milli-Q water in a 50 mL centrifuge tube. The mixture was shaken for 2 h at 22.5 °C to ensure thorough extraction. Afterwards, the sample was centrifuged at 4000 rpm for 10 min to separate solid particles. The supernatant was filtered through a 0.45 µm glass microfibre filter to remove any residual particulate matter. Samples were acidified with 1% nitric acid (HNO_3_) before the measurements. Standards for calibration were prepared from 10 ppm stock solutions (Merck Supelco®, 1000 mg/L of S or Fe, in HNO_3_) to achieve final concentrations of 7.5 ppm, 5 ppm, 2.5 ppm, 1 ppm, 0.5 ppm, and 0 ppm. If necessary, samples were further diluted to bring their concentrations within the calibration range.

#### Exoenzyme potential activities (µmol product/g DW/h) and pH

The exoenzyme potential activities (PAs) were monitored due to their role in decomposition processes. The activity of the β-d-glucosidase (GLU) enzyme is used as a proxy for the decomposition of cellulose, which is abundant in peat due to its plant origin (Sinsabaugh and Follstad Shah 2011). The arylsulfatase (SUL) enzyme breaks down organic sulphur compounds and releases sulphate, which plays a direct role in sulphur cycling (Tabatabai and Bremner 1970). The phosphatase (PHO) enzyme, on the other hand, can be influenced indirectly by the presence of iron, as it alters the availability of inorganic phosphate (Sinsabaugh and Follstad Shah 2011).

The peat samples were analysed for the potential activity of these three hydrolytic exoenzymes by using the fluorogenic 4-methylumbelliferone (MUF) assay before and after the incubation experiment (adapted from Dunn et al. 2014). The samples were incubated with a 1 mM solution of the relevant substrate (Sigma-Aldrich®, USA) in stirring Turaxx tubes (IKA®-Werke GmbH & Co. KG). After incubation, the slurry was transferred to 2 mL Eppendorf tubes and centrifuged at 14,000 rpm for 5 min. The supernatant was then measured using a fluorescence spectrometer with black-bottom 96-well plates (Thermo Fisher Scientific Inc.®), with excitation at 360/40 nm and emission at 460/40 nm. Oxic samples were measured under laboratory conditions with approximately 20% oxygen, while anoxic samples (only for permAn samples) were measured in an anoxic chamber (Vinyl Anaerobic Chamber, Type A, Coy Laboratory Products, Inc., USA).

Changes in pH were measured in a demi-water extract using a Sentix 41 pH electrode (WTW GmbH, Weilheim, Germany), both before and after the experiment. Five grams of peat were mixed with 30 mL of Milli-Q water and shaken for two hours at 22.5 °C. The samples were then centrifuged at 4000 rpm for 10 min, and the supernatant was used to measure pH.

### Data analysis

All statistical analyses and visualisations were performed using R programming (R Core Team 2025). A generalised additive model (GAM) was applied to investigate the effects of ferric iron (Fe^3+^) and sulphate (SO_4_^2−^) treatments on carbon dioxide (CO_2_) production rates. The analysis included data from Fe^3+^ amendment, SO_4_^2−^ amendment, and control, with the dataset divided into anoxic and oxic subsets to avoid dependent values.

For the anoxic subset, the GAM model included “treatment” and “cycle” as fixed effects, with random effects accounting for variability at the “site” and “replicate” levels. To address potential autocorrelation in the time series data and between Cy1 and Cy2, a lag term was introduced, incorporating the previous value of the response variable as a predictor in the statistical model (Online Resource Eqs. 1–3, Tables S2–S4).

For the oxic subset, the ASD and ROU sites were analysed separately due to significant site differences. The ASD data were analysed using the same GAM structure as the anoxic subset, excluding “site” as a random effect. In contrast, for ROU, CO_2_ production rates in Cy1 and Cy2 differed significantly. Therefore, separate models were fitted for each cycle, with “treatment” as a fixed effect, “time” included as a smooth function with a lag term, and “replicate” as a random effect.

Linear models followed by Type II ANOVA were used to compare exoenzyme potential activities, pH, and water-soluble iron and sulphur concentrations before and after the incubation experiment. When parts of the dataset did not meet the assumptions of parametric tests, transformations were applied. If the assumptions could not be satisfied through transformation, non-parametric Kruskal–Wallis tests were conducted instead. Pairwise comparisons were performed to identify significant differences between groups.

## Results

### Carbon dioxide emissions over time

#### Anoxic measurements

**Fig. 2:**
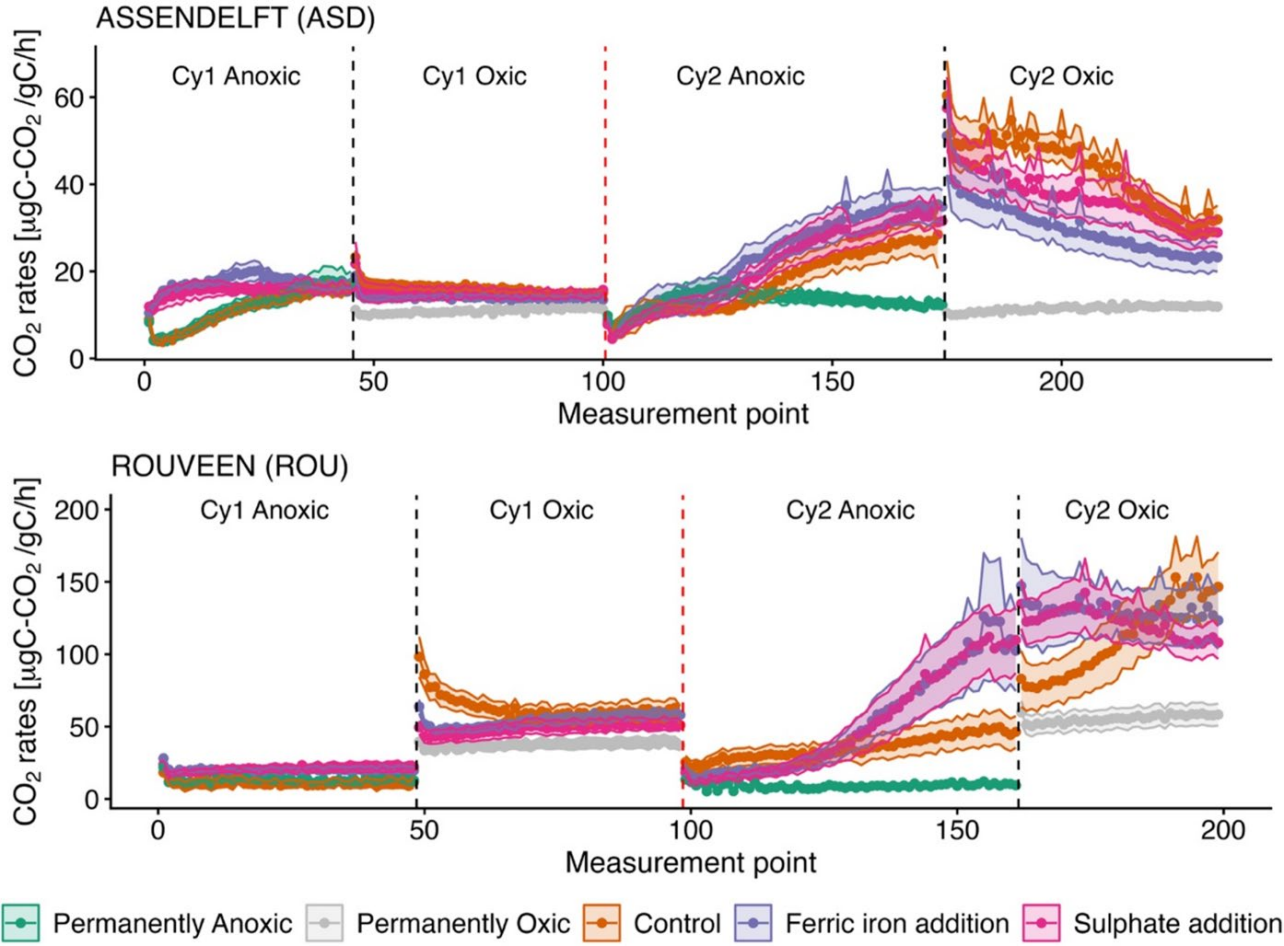
Laboratory measurements of CO_2_ emission rates from Assendelft and Rouveen peat samples (n = 6 per treatment); coloured lines represent means, and shaded bands indicate standard deviation. The x-axis represents measurement points taken with respirometers over a period of 5 to 7 days. Although labelled as sequential points (0, 1, 2,…,n), the actual time intervals between measurements vary between 90 and 140 min due to the capacity of the respirometer. Black vertical dashed lines indicate the oxygenation period (≈ 90 min), achieved by opening the lids and switching to the oxic respirometer. Red vertical dashed lines mark the time points at which samples return to the anoxic chamber for 72 h before starting the second cycle with lactate addition. PermAn samples were measured only under anoxic conditions, and PermOx samples were measured only under oxic conditions

Under anoxic conditions, ferric iron (Fe^3+^) and sulphate (SO_4_^2−^) amended samples were significantly different from the controls in terms of carbon dioxide (CO_2_) emissions (Fig. 2). This difference was more pronounced with Fe^3+^ amendment (p < 0.001, GAM) than with SO_4_^2−^ amendment (p < 0.1, GAM). The sites differed significantly in their response rates to Fe^3+^ and SO_4_^2−^ amendments, but with a clear increase in CO_2_ emission rates at both sites (p < 0.001, GAM). In the Rouveen (ROU) samples, Fe^3+^ and SO_4_^2−^ amendments resulted in a similar magnitude of increase in CO_2_ emission rates. However, in the Assendelft (ASD) samples, Fe^3+^ amendment resulted in a stronger increase of CO_2_ rates compared to controls (Fig. 2; Table S5). There was substantial heterogeneity among the field replicates (p < 0.1, GAM).

Cycle 1 (Cy1) and cycle 2 (Cy2) resulted in different CO_2_ emission rates, with higher emissions observed in the lactate-supplemented Cy2 (Fig. 2; Table S5). At Cy1, the control samples and permanently anoxic samples (permAn) behaved similarly [with average cumulative (avg. cum.) CO_2_ emissions of 424 and 442 µg C-CO_2_/g C, respectively, at the ASD site and 465 and 577 µg C-CO_2_/g C, respectively at the ROU site]. In contrast, during Cy2, permAn samples exhibited a consistently low CO_2_ emission rate under anoxic conditions, particularly at the ROU site compared to the control (Table S5). The only exception was the ASD permAn samples at Cy2, whose emission rates were initially comparable to the controls for approximately the first third of the experiment, after which they stabilised at a lower level. The highest avg. cum. CO_2_ concentrations from anoxic measurements were observed in Fe^3+^-amended samples during Cy2 at both sites (1575 µg C-CO_2_/g C for ASD and 2790 µg C-CO_2_/g C for ROU).

Compared to the controls, Fe^3+^ amendments resulted in an increase in average cumulative CO_2_ emission rates of 64% in Cy1 and 36% in Cy2 at the ASD field site. At the ROU site, the increases were 85% and 40% for Cy1 and Cy2, respectively. In contrast, SO_4_^2−^ amendments led to smaller increases at the ASD site (46% in Cy1 and 20% in Cy2). However, at the ROU site, sulphate amendment had a greater effect, with cumulative CO_2_ emission rates 91% higher than the control at the end of the Cy1 anoxic incubation. This difference declined to 35% in Cy2. Overall, the differences in CO_2_ emissions between the control and amended samples were smaller in the second cycle (Table S6).

#### Oxic measurements

In the oxic dataset, site responses, particularly in Cy2, differed significantly. Therefore, the sites were analysed separately. For the ASD oxic samples, the presence of Fe^3+^ and SO_4_^2−^ significantly reduced CO_2_ emission rates compared to the controls (Fig. 2). However, this difference diminished over time. The replicates exhibited high heterogeneity, and Cy2 differed significantly from Cy1 (p < 0.01, GAM).

For the ROU samples, the effects of Fe^3+^ and SO_4_^2−^ amendments followed similar pattern to ASD samples in Cy1 but behaved different in Cy2. At the ROU site in Cy2, Fe^3+^- and SO_4_^2−^-amended samples initially produced higher CO_2_ levels, but after a few days, the control samples surpassed them. Another notable difference was that replicates were more homogeneous in the ROU samples than in those from ASD.

Across all sites and cycles, the permanently oxic (permOx) samples consistently exhibited the lowest CO_2_ production rates compared to both the controls and amended treatments. In the oxic dataset for Cy2, control samples from ASD accumulated the highest CO_2_ levels (avg 2500 µg C-CO_2_/g C), while among the ROU samples, Fe^3+^-amended treatments had the highest accumulation (avg 4300 µg C-CO_2_/g C).

Under oxic conditions at the ASD site, Fe^3+^ and SO_4_^2−^ amendments led to 14% and 7% lower cumulative CO_2_ emissions than the control in Cy1, respectively. In Cy2, Fe^3+^ and SO_4_^2−^ amendments continued to reduce CO_2_ emissions compared to the control, with 32% and 15% lower emissions, respectively, while the permOx treatment caused a 74% reduction. At the ROU site, Fe^3+^ and SO_4_^2−^ amendments in Cy1 led to 15% and 24% lower CO_2_ emissions compared to the control, respectively. In Cy2, Fe^3+^ and SO_4_^2−^ treatments increased emissions by 23% and 17%, respectively, whereas the permOx treatment still reduced CO_2_ emissions by 48% relative to the controls (Table S6).

### Concentrations of water-soluble iron and sulphur

**Fig. 3:**
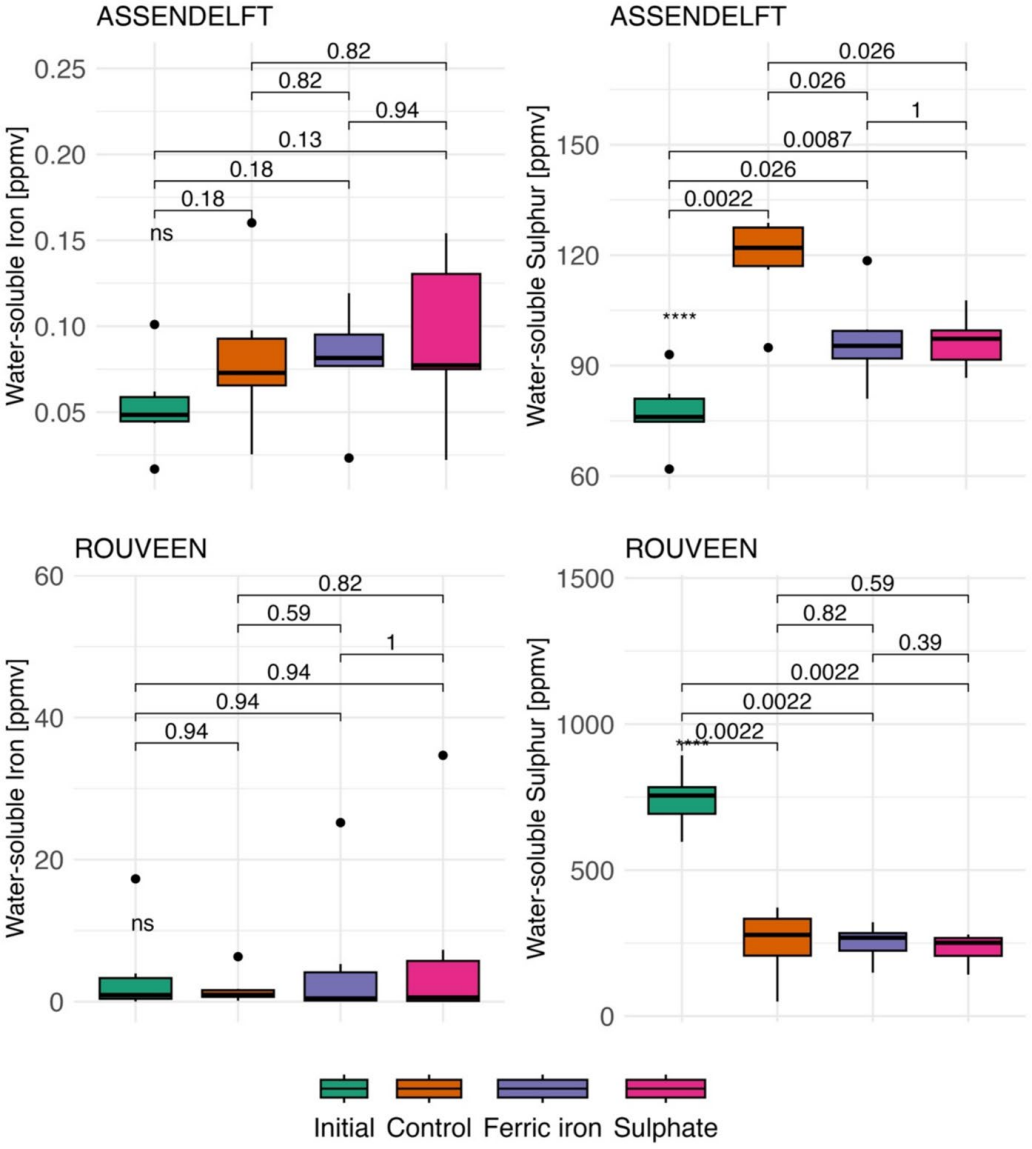
Water-soluble iron and sulphur concentrations in peat samples (ppm by volume—ppmv) across different conditions: initial values (before the experiment), experiment controls, and treatments with Fe^3+^ and SO_4_^2−^ amendments (after the experiment). ANOVA was applied for statistical analysis, except for iron concentrations of Rouveen samples, where the Kruskal–Wallis non-parametric test was used (***p < 0.01)

Water-soluble iron concentrations did not exhibit a significant change with Fe^3+^ amendment (Fig. 3). In the ASD and ROU samples, iron concentrations were similar in both the control and treatment groups (p > 0.1, ANOVA). In addition, their concentrations were not significantly different from the initial water-soluble iron concentrations (p > 0.1, ANOVA). ROU samples had relatively higher water-soluble iron concentrations than ASD samples at the end of the incubation. Water-soluble sulphur concentrations, on the other hand, showed significant variation. Initial sulphur concentrations were lower in the ASD samples (avg. 88.9 ± 11.7 ppmv) and higher in the ROU samples (avg. 803.0 ± 96.3 ppmv), compared to the controls (avg. 136.0 ± 14.6 ppmv and 169.0 ± 33.5 ppmv, respectively) after the experiment (p < 0.01, ANOVA). While the ROU control and treatment samples had similar water-soluble sulphur concentrations, the ASD treatments (both Fe^3+^ and SO_4_^2−^amended samples) exhibited lower concentrations compared to the controls (Fig. 3). Both control and treatment samples were significantly different from the initial water-soluble sulphur concentrations (p < 0.01, ANOVA).

The permanently anoxic samples (permAn) from both sites showed the highest water-soluble iron concentrations (avg. 8.4 ± 5.1 ppmv for ASD and 15.5 ± 12.9 ppmv for ROU), while no significant differences were observed in the permanently oxic samples (permOx) compared to the treatments and controls (Fig. S1). Water-soluble sulphur concentrations, however, were significantly higher in the permOx samples from the ASD site (avg. 181.6 ± 15.6 ppm). In contrast, the permAn samples (avg. 97.1 ± 22.2 ppmv) showed similar values compared to the treatments (avg. 96.7 ± 9.8 ppmv). For the ROU site, the permOx samples had relatively high water-soluble sulphur concentrations (avg. 327.9 ± 76.0 ppmv), but these were not significantly different from the controls and treatments (avg. 245 ± 78.4 ppmv). The permAn sample (avg. 135.7 ± 43.0 ppmv) had relatively low concentrations, but both the permOx and permAn concentrations were significantly lower than the initial concentrations (p < 0.1, ANOVA).

### Exoenzyme potential activities and pH

**Fig. 4:**
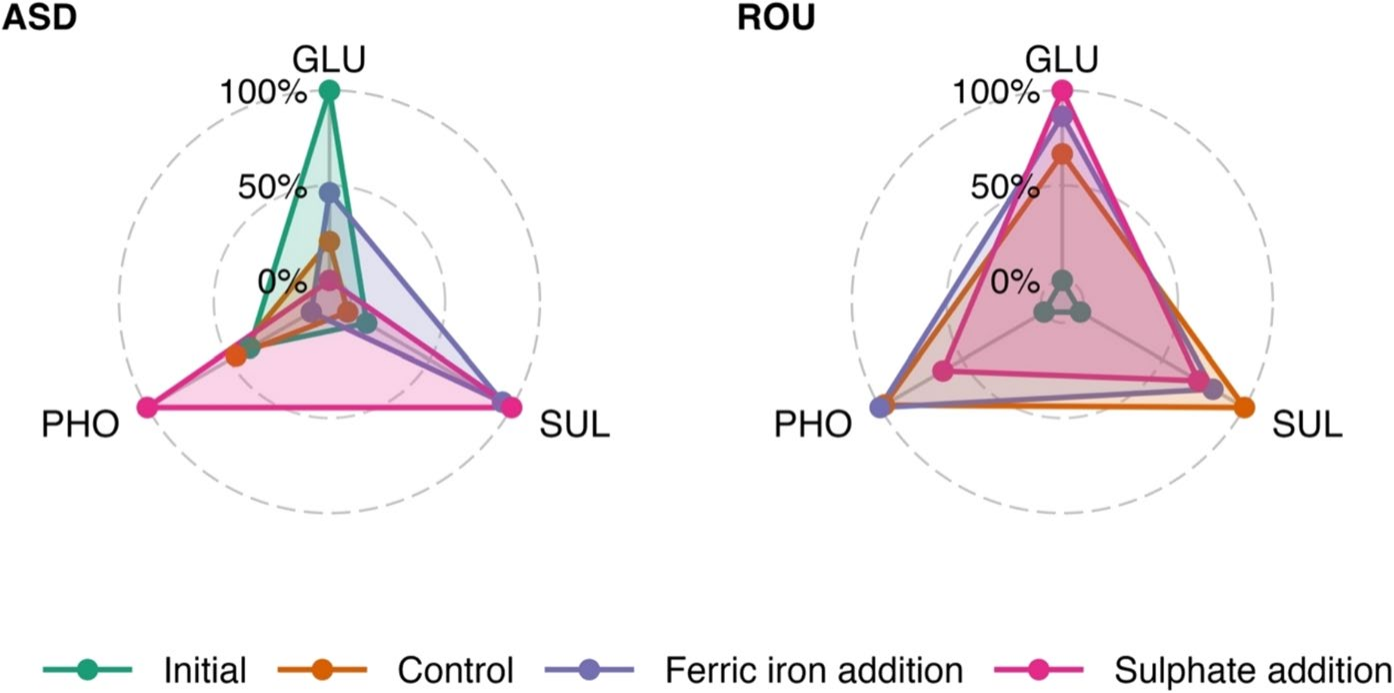
β-d-glucosidase (GLU), arylsulfatase (SUL), and phosphatase (PHO) activities for initial, control, Fe^3+^ amendment, and SO_4_^2−^ amendment samples from the Assendelft (ASD) and Rouveen (ROU) sites. For each site, activities were independently normalized to a 0–100 scale, where 0 and 100 represent the minimum and maximum observed activity across all treatments within that site. Values therefore reflect treatment-specific differences relative to the site-specific activity range and are not directly comparable between sites

The amendment experiment altered the exoenzyme potential activities (PAs) of β-d-glucosidase (GLU), arylsulfatase (SUL), and phosphatase (PHO) compared to controls, but the differences were not significant at both sites. However, the exoenzyme PAs of the incubated samples were significantly different from the initial activities in ROU samples (ANOVA p < 0.01, Fig. 4).

β-d-glucosidase PA in ASD samples was not significantly different overall, except for the permAn and permOx treatments (Table 3, ANOVA followed by pairwise comparison p < 0.01). The highest GLU PA was observed in the permAn samples (avg. 2.33 µmol/g/h) while the lowest was recorded in the permOx samples, with an average of 0.95 µmol/g/h. In Rouveen samples, a similar trend was observed. The permAn treatment exhibited the highest GLU PA (avg. 4.67 µmol/g/h) and the permOx treatment had the lowest GLU PA (avg 0.87 µmol/g/h). Initial GLU PA in Rouveen was lower, with an average of 0.96 µmol/g/h.

**Table 3:** Exoenzyme PAs and pH values before and after the incubation experiment [β-d-glucosidase (GLU), arylsulfatase (SUL), and phosphatase (PHO) all expressed in µmol-product/g/h ± standard deviation (sd)]

Field site	Treatment	pH	GLU	SUL	PHO
Assendelft (ASD)	Field/Initial	4.9 ± 0.3	1.47 ± 0.26	0.22 ± 0.14	7.84 ± 1.70
	Control	4.8 ± 0.3	1.32 ± 0.30	0.19 ± 0.12	7.95 ± 3.76
	Fe^3+^ amendment	4.7 ± 0.3	1.37 ± 0.18	0.41 ± 0.08	7.36 ± 3.21
	SO_4_^2−^ amendment	4.7 ± 0.3	1.28 ± 0.30	0.42 ± 0.04	8.65 ± 4.75
	permAn	4.8 ± 0.3	2.33* ± 0.88	0.25 ± 0.36	7.33 ± 1.38
	permOx	4.0 ± 0.2	0.95* ± 0.32	0.04* ± 0.06	5.64 ± 1.50
Rouveen (ROU)	Field/Initial	3.7 ± 0.3	0.96 ± 0.28	0.04* ± 0.05	1.90 ± 0.88
	Control	4.0 ± 0.4	2.02 ± 0.53	0.40 ± 0.17	6.03 ± 2.40
	Fe^3+^ amendment	3.7 ± 0.3	2.34 ± 0.54	0.33 ± 0.04	6.14 ± 1.76
	SO_4_^2−^ amendment	3.8 ± 0.4	2.55 ± 0.88	0.30 ± 0.07	4.52 ± 1.14
	permAn	4.3 ± 0.1	4.67* ± 1.81	0.71* ± 0.32	10.36* ± 2.60
	permOx	3.2 ± 0.4	0.87 ± 0.16	0.20 ± 0.18	2.06 ± 0.74

*p < 0.01 samples significantly different than all groups (ANOVA followed by pairwise comparison or Kruskal*–*Wallis followed by Post-Hoc Dunn test with Bonferroni correction)

Arylsulfatase PA in ASD samples showed increases with Fe^3+^ and SO_4_^2−^ amendments, (avg. 0.41 and 0.42 µmol/g/h, respectively), compared to the initial value of 0.22 µmol/g/h (ANOVA followed by pairwise comparison p < 0.01). The lowest SUL PA was observed in permOx samples (avg. 0.04 µmol/g/h). In Rouveen samples, SUL PA was generally low, with the highest activity in the permAn treatment and the lowest under initial conditions.

Phosphatase PA in ASD samples was relatively stable across treatments, with a slight increase observed under SO_4_^2−^ amendment (avg. 8.65 µmol/g/h), compared to initial values (avg. 7.84 µmol/g/h). The permOx samples exhibited the lowest PHO PA. In contrast, ROU samples showed much lower initial PHO PA, which increased substantially in the permAn treatment (avg. 10.36 µmol/g/h, Kruskal–Wallis followed by Post-Hoc Dunn test with Bonferroni correction, p < 0.001). The permOx treatment in Rouveen had the lowest PHO PA.

After two cycles and the amendments of Fe^3+^ and SO_4_^2−^, the pH of the treatment and control samples was not significantly different from the Fe^3+^ and SO_4_^2−^-amended samples (Table 3, p > 0.1, ANOVA). For ASD samples, the highest pH was measured under initial conditions, with an average pH of 4.9, while the lowest pH was recorded in the permOx samples after the experiment, with an average pH of 4.0. In Rouveen samples, the highest recorded pH was 4.3, observed in samples kept anoxic throughout the experiment (permAn). The lowest pH values in Rouveen were recorded in the permOx samples, with an average pH of 3.2 after the experiment.

## Discussion

Our results showed that the increased concentrations of ferric iron and sulphate altered the rates of carbon dioxide (CO_2_) emissions both under anoxic and oxic conditions at the transition zone from drained coastal peatlands in the Netherlands. While CO_2_ emission rates increased with ferric iron (Fe^3+^)‐ and sulphate (SO_4_^2−^)‐amendment under anoxic conditions, there were short but significant impacts on the reduction of CO_2_ emission rates under oxic conditions as we hypothesised.

### Carbon dynamics of anoxic transition zone

Under high groundwater-table conditions, the transition zone becomes saturated after an oxic period. Oxidised ions are brought in, which supports anaerobic microbial activity. Our data from cycle 1 (Cy1) anoxic measurements revealed that increasing concentrations of ferric iron (Fe^3+^) and sulphate (SO_4_^2−^) ions in samples collected from the transition zone significantly increased CO_2_ emission rates during anoxic measurements. This increase can be linked to enhanced heterotrophic respiration via sulphate-reducing bacteria (SRB; Anantharaman et al. 2018; Blodau et al. 2007; Hausmann et al. 2018; Pester et al. 2012) and iron-reducing bacteria (IRB; Ionescu et al. 2015; Küsel et al. 2008; Li et al. 2021; Van De Velde et al. 2020; Wang et al. 2017) which use Fe^3+^ and SO_4_^2−^ as alternative terminal electron acceptors (ATEAs). However, the response over time to added ATEAs differed between sites.

For Assendelft site (ASD), there is a clear and sharp response to amendments, with increased CO_2_ emission rates at the start of the anoxic measurements. A similar response is observed in controls and permanently anoxic (permAn) samples, but at lower rates. After the added ATEAs are consumed in the amended samples, CO_2_ emission rates become similar, indicating that other factors, such as substrate quality (Preston et. al. 2012), may limit microbial activity.

The response in controls and permAn samples in Cy1 suggests that the ASD site already contains oxidised ATEAs during the oxic phase of the transition zone. When the samples were placed in an anoxic environment, these oxidised ions supported the anaerobic community to respire at rates comparable to those seen in subsequent oxic measurements. The high CO_2_:methane (CH₄) ratio reported in the porewater of ASD samples (104.6 ± 94.5, n = 20, high water table period at 45 cm depth; NOBV Database) suggests that most of the microbial community at this site relies on CO_2_ metabolic pathways. This value supports the response curve observed at the start of the anoxic measurements in Cy1. Long-term porewater chemistry data also show that ASD is rich in sulphur, sulphide, PO_4_, NH_4_, and NO_3_ ions compared to ROU, indicating that the microbial community is already adapted to this ion rich environment and utilises these ATEAs to degrade organic matter under anoxic conditions (Table S1). The results of this experiment together with pore-water chemistry data support the battery charging hypothesis for the ASD site.

The response over time under anoxic conditions for Rouveen site (ROU), on the other hand, was more stable compared to ASD in Cy1. This may be due to competition between SRB, IRB, and methanogens. In anoxic environments, methanogens compete with SRB and IRB for substrates such as acetate and hydrogen. The presence of SO_4_^2−^ and Fe^3+^ intensifies this competition, favouring CO_2_ production over CH_4_ (Achtnich et al. 1995; Boothroyd et al. 2021; Chou et al. 2008; Knorr et al. 2009; Koebsch et al. 2019; Lovleyt and Klug 1983). In Cy1 of the experiment, stable CO_2_ emission rates in ROU samples compared to ASD samples might suggest that the microbial community took advantage of the additional electron acceptors without entirely outcompeting methanogens in ROU samples. Additionally, some of the produced CO_2_ may have been consumed by methanogens, potentially stabilising the net CO_2_ emissions under anoxic conditions during the measurement period (Lehmann‐Richter et al. 1999; Thiel et al. 2019). At the ROU site, much lower CO_2_:CH_4_ ratio in the porewater (12.3 ± 2.9, n = 10 high water table period at 45 cm depth; NOBV Database) was reported indicating more abundance of methanogens. In addition, archaeal groups such as Methanosarcinia, Methanoperedenaceae, and Hadarchaeales were detected in the ROU anoxic zone compared to ASD anoxic zones further supports the competition hypothesis (Tolunay 2025).

The results from ASD and ROU under anoxic conditions (Cy1) indicate that the fluctuating water table in the transition zone plays a more critical role in the carbon cycle of drained peatlands with ion-rich (ATEAs) porewater chemistry.

In Cycle 2 (Cy2), lactate addition removed substrate limitation. Additionally, the amended samples experienced no limitation of ATEA, which significantly boosted CO_2_ emission rates at both sites. Although the response to amendments was similar, CO_2_ emission rates were considerably higher in ROU samples, indicating that this site was substrate limited. The lower carbon density data (Table 2) further supports this interpretation (Luo et al. 2025). Lactate addition, together with the amendments in the ROU samples, may have also favoured microbial species that rely on CO_2_-based metabolic pathways, such as SRB and IRB, allowing them to outcompete methanogens. This could have enhanced their growth rates and led to their dominance in ROU during Cy2, which may explain the exponential CO_2_ emission rates curve observed in the amended samples compared to the controls (Kotsyurbenko et al. 2004; Vile et al. 2003a, 2003b). In the controls, the ASD site showed a similar trend to the amended samples but with lower CO_2_ emission rates, while ROU samples displayed a slower but steady increase over time. This clearly indicates that the dominant metabolic pathways under anoxic conditions are governed by both substrate and ATEA limitations.

Carbon dioxide emission rates initially followed similar patterns in both permanently anoxic (permAn) and control (Con) samples; however, they diverged over time (in Cy2) due to depletion of electron donors and acceptors in permAn samples (Megonigal et al. 2003; Sutton-Grier et al. 2011). The ASD permAn samples in Cy2 initially produced similar rates of CO_2_ emissions compared to controls, but the rate then decreased. Sustained high CO_2_ emission rates at the beginning of the Cy2 from permAn samples might indicate that the ASD samples could still provide sufficient substrate and ATEAs after long-term anoxic incubation, highlighting the importance of peat material not only as a source of substrate but also as a potential source of ATEA under harsh environmental conditions.

Overall, the anoxic dataset indicates that the activity of the anaerobic microbial community in the transition zone of drained coastal peatlands is shaped by the availability of electron acceptors and substrates. This may lead to increased total carbon fluxes during periods of high-groundwater table.

### Carbon dynamics of oxic transition zone

During low groundwater table periods, oxygen begins diffusing into the transition zone, creating oxic and anoxic pores and gradually shifting microbial activity towards aerobic pathways over time. For ASD samples in Cy1, transition from anoxic to oxic did not significantly change the range of CO_2_ emission rates. In ROU site however, the CO_2_ emission rates under oxic conditions were significantly high compared to anoxic measurements. This supports the hypothesis that the substrate of ROU is difficult to degrade under anoxic conditions and requires aerobic, more energy efficient pathways. Both electron acceptors and substrate seem limiting to ROU decomposition rates.

The oxic dataset from both sites in Cy1 showed clear lower CO_2_ emission rates within the first few hours of oxygen exposure in the amended samples compared to the controls. A potential explanation is that obligate anaerobic communities may have sharply declined upon oxygen exposure. Since the abundance of anaerobic species was likely higher in the amended samples, the impact would have been more pronounced. However, as oxygen availability increased, the microbial community may have shifted toward aerobic organisms, leading to the use of more energy-efficient aerobic metabolic pathways. (Huang et al. 2021, 2023). This, in turn, diminished the impact of the amendments in the samples. In addition to the community shift, the observed reduction in CO_2_ emission rates, particularly in the Fe-amended samples compared to the controls, may be attributed to the oxidation of Fe^2+^, which might have led to the precipitation of insoluble iron hydroxides [e.g., Fe(OH)_3_]. The formation of these compounds likely reduced the availability of Fe^3^⁺ as an electron acceptor for iron-reducing bacteria (IRB) in the anoxic pore environments (Riedel et al. 2013). Moreover, under oxic conditions, Fe^2+^ oxidation to Fe^3+^ generates reactive oxygen species (ROS), such as hydroxyl radicals (OH^·^) and superoxide anions (O_2_^−^), which can damage microbial exoenzymes and further lower CO_2_ emissions (Zhao et al. 2021). However, we did not observe any significant changes in exoenzyme potential activities, which argues against the latter explanation. Another possible explanation is that iron oxides can adsorb DOC, limiting access to readily available carbon in the short term (Wu et al. 2008). The decrease in CO_2_ emission rates also aligns well with the *Iron Gate* hypothesis, which suggests that Fe–lignin associations might act as protection against the decomposition of organic matter (Wang et al. 2019; Wen et al. 2019). However, this effect did not persist for more than a week, and CO_2_ emission rates were almost identical between treatments and controls by the end of the experimental period, with similar pH values.

At ROU, oxic Cy2 samples exhibited a unique trend. Initially, higher CO_2_ emission rates were observed in the Fe^3+^ and SO_4_^2−^ treatments compared to the controls. Later in the measurement period, CO_2_ emission rates became higher in the controls compared to treatment samples. This pattern suggests that labile carbon (added lactate) remained in the control samples because the anaerobic community did not grow as extensively as in the amended ones. As a result, the remaining lactate may have been used by the aerobic community over time during oxic phase (Moazeni et al. 2010; Schönheit et al. 1982).

Permanently oxic (permOx) samples had the lowest CO_2_ emissions, reflecting electron donor and acceptor exhaustion like in permAn samples. This has also been shown by long-term laboratory incubations (Weidner et al. 2022). Peat decomposition can be very rapid at the beginning of oxygenation, but over time, easily decomposable materials decrease, and more time-consuming pathways are required to access the necessary substrates for microbial activity and growth. These results confirm that oxygen alone does not drive decomposition; substrate also plays critical role in determining the decomposition rates (Kravchenko et al. 2019; Shannon and White 1996; Strong et al. 2004).

Although several findings in literature suggest that iron can promote organic matter degradation during drought via the precipitation of phenols (Hall et al. 2014; Wang 2019; Wang et al. 2022a, b), our short-term results do not support this hypothesis. Therefore, long-term measurements, disentangling the effects of electron acceptors and donors would be a worthwhile next step in this research.

In summary, oxygen intrusion into the transition zone, where iron and sulphur species are in higher concentrations, can temporarily limit decomposition rates. Furthermore, our data show that anaerobic processes can influence the activity of aerobic communities in the transition zone. This connection should not be overlooked when quantifying carbon fluxes from managed, drained peatlands.

### Concentrations of water-soluble iron and sulphur

Our results showed no significant change in water-soluble iron concentrations in either ASD or ROU samples after the experiment. This stability likely reflects the lack of leaching in our setup and the low solubility of Fe^3+^ under oxic conditions. Since bioavailable iron makes up only a small fraction of total iron (Chen et al. 2020; Riedel et al. 2013), and Fe–organic matter complexes can sequester both iron and dissolved organic carbon (Huang et al. 2021; Zhao et al. 2019), water extraction alone may underestimate availability.

In contrast, initial sulphur levels were unexpectedly higher in ROU than in ASD, despite field measurements showing the opposite (Boonman 2024). This discrepancy may reflect seasonal differences: ROU was sampled after summer drought, while ASD sampling followed autumn rains that may have induced micro-anoxic conditions and altered sulphur levels. After the experiment, ASD treatments showed a marked decrease in water-soluble sulphur, suggesting enhanced mineralisation or reduction (Hockin and Gadd 2003), while ROU samples showed little change despite distinct CO_2_ patterns. This might lay in the difference in anaerobic microbial community. Reduced sulphur might have been in different species depending on the metabolic pathways of the microbial community (i.e. assimilatory sulphate reduction) or abiotic processes (i.e. pyrite formation) can alter the forms of the reduced sulphur species (Schiff, 1980). This can lead to less H_2_S_(g)_ production and therefore less lost from the system in ROU samples.

While informative, water-soluble measurements alone cannot fully capture the complex redox-driven processes behind CO_2_ emissions in iron- and sulphur-rich peat. Future work should assess total and bioavailable pools, as well as speciation of Fe and S, to better understand these dynamics.

### Exoenzyme potential activities and pH

The results of potential activities (PAs) of key exoenzymes, β-d-glucosidase (GLU), arylsulfatase (SUL), and phosphatase (PHO) varied with redox conditions, reflecting the sensitivity of soil biochemical processes to oxygen and pH (Bonnett et al. 2017; Wang et al. 2022a, b). Across both sites, exoenzyme PAs were generally higher under anoxic conditions. Notably, GLU activity peaked in permAn samples, likely due to the depletion of electron donors and acceptors driving microbes to increase enzyme production (Riedel et al. 2013). This challenges the first part of the *Enzymic Latch* hypothesis, which predicts suppression of hydrolytic enzymes under anoxia (Freeman et al. 2001). permOx samples showed lower GLU activity, potentially due to pH declines controlling enzyme function (Huang et al. 2023) and reduced demand for exoenzymes under aerobic conditions (Chen et al. 2020). Arylsulfatase and PHO activities showed site-specific responses, but their activities were not correlated with the addition of alternative electron acceptors. Although we attempted to measure oxidative enzymes (e.g. phenol oxidase—POX), interference between iron and the l-3, 4-dihydroxyphenylalanine (l-DOPA) substrate compromised results (Campbell et al. 1989; Hamada and Rogers 2007; Linert et al. 1991). Given similar issues with 2, 2′-azino-bis(3-ethylbenzothiazoline-6-sulfonic acid) (ABTS) substrate (Luo et al. 2021), we excluded POX data and recommend caution when using these substrates in iron-rich systems.

Enzyme PAs correlated more strongly with pH than redox state. Low pH reduced activity, suggesting acidity plays a greater role than redox mechanisms (Cornish-Bowden 1979; Li et al. 2020). However, since exoenzyme data were collected post-incubation, their activities under anoxic conditions may have differed. Overall, exoenzyme PAs appear more relevant to nutrient cycling and stoichiometry than to microbial respiration potential in small scale experiments.

### Implications of redox dynamics on carbon dioxide emissions in drained coastal peatlands

Our experiment captured a snapshot of redox changes in the transition zone, where the groundwater table regularly fluctuates. Regarding the experimental setup, our samples were maintained under optimal laboratory conditions (22.5 °C), which likely resulted in CO_2_ emissions higher than those observed in the field. However, with an increasing trend in soil warming due to prolonged warm periods in temperate climates (Soong et al. 2020), our results may also provide insight in potential future trends in CO_2_ emissions.

It is important to note that this was a small-scale, short-term experiment (maximum duration of 1 week per cycle), and responses may vary under longer-term environmental changes. Although our study captured short-term responses, as extreme climatic events become more frequent, understanding these transient responses will be critical for a comprehensive understanding of carbon dynamics in peatland ecosystems.

Current models of greenhouse gasses (GHG) for peatlands largely overlook the role of the transition zone and assume that CO_2_ emissions originate primarily from the oxic layer above the groundwater table, applying fixed, homogeneous emission rates throughout the oxic depth (Wilson et al. 2016). These models ignore anaerobic microbial activity and treat the transition zone as either fully oxic or irrelevant, despite its potential to contribute significantly to total CO_2_ emissions under varying groundwater-table conditions. Our findings challenge the traditional view by showing that microbial activity in the transition zone responds dynamically to changes in electron donor and acceptor availability. Unlike the permanently anoxic or oxic zones, the transition zone acts like a charging battery, enabling it to contribute disproportionately to emissions compared to other layers. This implies that the entire peat profile is not equally active. Thus, limiting models to the oxic zone alone significantly underestimates or overestimates total emissions. Including the transition zone is therefore essential for improving carbon flux estimates. While previous studies have acknowledged the importance of the full peat profile (Blodau et al. 2007; Gauci et al. 2021; Keller & Takagi 2013; Lamers et al. 2002), our work builds on this by explicitly disentangling the roles of the transition zone versus the permanently anoxic and oxic zones, highlighting the distinct and dynamic contribution of redox-sensitive layers. Given that ditch water levels in Dutch peatlands may be supplied with brackish water in the future, these findings also provide valuable insights into how iron and sulphur might alter carbon dynamics. Besides iron and sulphur, brackish water management and sea-level rise also introduce other ions into the system, such as sodium (Na^+^), chloride (Cl^−^), magnesium (Mg^2+^), and calcium (Ca^2+^) (Brouns et al. 2014; Canavan et al. 2006). It is important to study these ions too, as well as their interactions with each other and with the peatland carbon cycling.

## Conclusions

In this study, we investigated the impact of iron (Fe^3+^) and sulphate (SO_4_^2−^) amendments, together with changes in substrate availability, on biogeochemical processes and carbon dioxide (CO_2_) emissions in drained coastal peatlands by focusing on the transition zones where groundwater table fluctuations are influenced by water management practices and climate change.

Under anoxic conditions, the presence of Fe^3+^ and SO_4_^2−^ significantly enhanced CO_2_ emission rates, potentially due to their role as alternative electron acceptors that stimulate heterotrophic anaerobic microbial activity. In contrast, under oxic conditions, these amendments appeared to suppress CO_2_ emissions in the short term, possibly due to the limited availability of labile organic carbon and shifts in the metabolic pathways of the microbial community. These results emphasise how alternating redox conditions can act like a battery: under oxic conditions, the “battery” is charged, and when anoxic conditions return, it is discharged by the anaerobic microbial community in drained coastal peatlands. The charged battery hypothesis was most evident at the site with abundant alternative electron acceptors. This suggests that future increases in these inputs, driven by seawater intrusion and brackish water management could complicate GHG emission estimates from drained coastal peatlands. Our findings provide insight into how these systems may respond to changing redox conditions in terms of carbon balance and GHG dynamics.

## Supplementary Information

Below is the link to the electronic supplementary material.

Supplementary file1 (DOCX 314 kb)

## Data Availability

The experimental data of this study are available in Utrecht University Yoda research data management service at the following URL: 10.24416/UU01-H11BUG.
