# Supplementary material for: Iron and sulphur regulate carbon dioxide emissions in drained coastal peatlands of The Netherlands
Source: Biogeochemistry. 2026 Jan 9;169(1):8. doi: 10.1007/s10533-025-01303-x (PMC12835034; doi:10.1007/s10533-025-01303-x)
Supplement: Supplementary file 1 — Supplementary file1 (DOCX 314 kb) [file 10533_2025_1303_MOESM1_ESM.docx]

**The Supplementary Information**

**For Submission to the Journal Biogeochemistry**

**Iron and Sulphur Regulate Carbon Dioxide Emissions in Drained Coastal Peatlands of the Netherlands**

Duygu Tolunay^a,^*, Gizem Ergut^a^, Levi Simon^a^, Gilles Erkens^a,b^, George A. Kowalchuk^a^, Mariet M. Hefting^a,c^

^a^*Ecology and Biodiversity, Institute of Environmental Biology, Utrecht University, Padualaan 8, 3584 CH Utrecht, the Netherlands*

^b^*Deltares Research Institute, P.O. Box 85467, 3508 AL Utrecht, the Netherlands*

^c^*Amsterdam Institute for Life and Environment (A-LIFE), Systems Ecology Section, Vrije Universiteit Amsterdam, Van der Boechorstraat 3, 1081 BT Amsterdam, the Netherlands*

**Corresponding author*

*E-mail address:* [d.tolunay@uu.nl](mailto:d.tolunay@uu.nl)

The supplementary file is provided to give detailed information on site characteristics and the statistical analyses, further clarifying our methodological approaches. Moreover, additional results are shared in the form of tables and figures to present more data.

The file includes details of the pore-water chemistry of the field sites, which were collected as part of another project. It also includes the Generalised Additive Model (GAM) equations and model outputs, which reveal the statistical significance of the tested hypotheses. Furthermore, we present total water-soluble Fe and S concentrations of permanently anoxic and permanently oxic samples. These were mentioned in the main manuscript, but to keep the figures concise and readable, they were not included in the manuscript. Averaged cumulative carbon dioxide emissions and the direction of changes in emissions of the treatments compared with the controls are also provided in the supplementary file.

**Table S1**. Pore-water chemistry of the experiment sites (For ROU n=85, For ASD n=185 measured between 2020 and 2022). Avg=Average, sd=standard deviation, Min=measured minimum value, Max= measure maximum value. ND=Not detectable (<<0.0001). Data was also split into two showing the difference in pore-water chemistry of transition zone under anoxic and oxic conditions depending on the water table depth. Data is provided by Netherlands Research Programme on Greenhouse Gas Dynamics in Peatlands and Organic Soils (NOBV): <https://www.nobveenweiden.nl/en/> . (DOC unit mmol/L, the rest in μmol/L, pH is unitless)

| Site |  | pH | NO_3_ | NH_4_ | PO_4_ | Fe | S | DOC | methane | sulfide |
| --- | --- | --- | --- | --- | --- | --- | --- | --- | --- | --- |
| ASD | **Avg** | 4.7 | 32.2 | 263.6 | 13.9 | 543.3 | 9726.0 | 13.5 | 12.9 | 1.1 |
|  | **sd** | 0.4 | 113.1 | 182.9 | 14.3 | 417.0 | 3506.0 | 6.5 | 20.0 | 2.6 |
|  | **Min** | 4.1 | ND | 0.9 | 0.1 | 9.9 | 967.2 | 2.6 | 0.2 | 0.02 |
|  | **Max** | 6.761 | 846.0 | 1055.9 | 66.1 | 1494.0 | 19988.3 | 34.0 | 218.6 | 17.5 |
|  | Avg values measured at depth 45 cm under **anoxic** conditions (high water table period n=20) | | | | | | | | | |
|  | **Avg** | 4.4 | 0.9 | 74.9 | 5.2 | 358.13 | 11421.7 | 10.4 | 10.5 | 1.2 |
|  | **sd** | 0.5 | 0.8 | 55.2 | 3.7 | 247.2 | 3769.8 | 3.1 | 12.7 | 1.2 |
|  | Avg values measured at depth 45 cm under **oxic** conditions (low water table period n=80) | | | | | | | | | |
|  | **Avg** | 4.7 | 72.8 | 226.2 | 7.0 | 253.3 | 8710.1 | 8.8 | 5.7 | 0.1 |
|  | **sd** | 0.4 | 164.3 | 225.2 | 12.1 | 317.8 | 3709.6 | 5.4 | 6.4 | 0.1 |
| ROU | **Avg** | 6.3 | 5.8 | 91.0 | 5.5 | 268.4 | 824.9 | 14.3 | 89.3 | 0.2 |
|  | **sd** | 0.2 | 23.8 | 73.2 | 7.8 | 123.3 | 793.5 | 3.9 | 71.8 | 0.4 |
|  | **Min** | 5.8 | 0.4 | 2.4 | 0.3 | 23.4 | 50.6 | 6.6 | 3.0 | 0.004 |
|  | **Max** | 6.7 | 155.2 | 451.0 | 67.5 | 626.9 | 3192.1 | 26.11 | 333.4 | 2.2 |
|  | Avg values measured at depth 45 cm under **anoxic** conditions (high water table period n=35) | | | | | | | | | |
|  | Avg | 6.2 | 2.0 | 58.4 | 3.8 | 278.5 | 834.9 | 13.2 | 90.2 | 0.08 |
|  | sd | 0.1 | 1.3 | 73.9 | 4.7 | 163.2 | 871.6 | 5.3 | 66.7 | 0.2 |
|  | Avg values measured at depth 45 cm under **oxic** conditions (low water table period n=50) | | | | | | | | | |
|  | Avg | 6.4 | 8.5 | 113.9 | 6.6 | 261.3 | 817.9 | 15.0 | 88.7 | 0.4 |
|  | sd | 0.2 | 30.8 | 64.0 | 9.3 | 86.5 | 743.1 | 2.6 | 75.9 | 0.6 |

**GAM for Anoxic Subset**

${{CO}_{2}}^{(t)}=\beta_{0}+\beta_{1}.Treatment+ f\left( Time \right)+\beta_{2}.Cycle+\beta_{3}.Lag{{CO}_{2}}^{(t-1)}+ u_{Site}+u_{Replicate}+\varepsilon$ *Eq(1)*

**Table S2.** Output of the GAM for anoxic dataset. Signif. codes: ‘***’ = 0.001, ‘**’ = 0.01, ‘*’ = 0.1

| **Parameters** | **Std. Error** | **t value** | **Pr(>\|t\|)** |
| --- | --- | --- | --- |
| *Fe^+3^ amendment* | 0.27 | 4.04 | 5.38e-05*** |
| *SO_4_^2-^ amendment* | 0.27 | 1.57 | 0.10* |
| *Cycle-Cy2* | 0.23 | 3.42 | 0.0006*** |
| *LagCO_2_* | 0.01 | 200.43 | <2e-16*** |
| **Smooth Terms** | **p value** |  | |
| *s(Time)* | <2e-16*** | **R-sq (adj):** | 0.94 |
| *s(Replicates)* | 0.0167* | **Deviance explained:** | 94.2% |
| *s(Site)* | 7.46e-07*** | ***n*:** | 4284 |

**GAM for Oxic Subset**

For ASD Site for both cycles together:

${{CO}_{2}}^{(t)}=\beta_{0}+\beta_{1}.Treatment+f\left( Time \right)+ \beta_{2}.Cycle+\beta_{3}.Lag{{CO}_{2}}^{(t-1)}+u_{Replicate}+\varepsilon$ *Eq(2)*

**Table S3.** Output of the GAM for oxic dataset of ASD site. Signif. codes: ‘***’ = 0.001, ‘**’ = 0.01, ‘*’ = 0.1

| **Parameters** | **Std. Error** | **t value** | **Pr(>\|t\|)** |
| --- | --- | --- | --- |
| *Fe^+3^ amendment* | 0.26 | -5.9 | 3.03e-09*** |
| *SO_4_^2-^ amendment* | 0.24 | -2.9 | 0.003** |
| *Cycle-Cy2* | 0.31 | 13.3 | <2e-16*** |
| *LagCO_2_* | 0.32 | 69.9 | <2e-16*** |
| **Smooth Terms** | **p value** | **R-sq (adj):** | 0.90 |
| *s(Time)* | <2e-16*** | **Deviance explained:** | 90.1% |
| *s(Replicates)* | 0.0004*** | ***n*:** | 2199 |

For ROU Site for each cycle modelled separately:

${{CO}_{2}}^{(t)}=\beta_{0}+\beta_{1}.Treatment+f\left( Time \right)+\beta_{2}.Lag{{CO}_{2}}^{(t-1)}+u_{Replicate}+\varepsilon$ *Eq(3)*

**Table S4.** Outputs of the GAM for oxic dataset of ROU site for Cy1 and Cy2. Signif. codes: ‘***’ = 0.001, ‘**’ = 0.01, ‘*’ = 0.1

| **ROU Oxic Cy1 Data** | | | |
| --- | --- | --- | --- |
| **Parameters** | **Std. Error** | **t value** | **Pr(>\|t\|)** |
| *Fe^+3^ amendment* | 0.60 | -7.2 | 1.02e-12*** |
| *SO_4_^2-^ amendment* | 0.66 | -10.1 | <2e-16*** |
| *LagCO_2_* | 0.03 | 22.1 | <2e-16*** |
| **Smooth Terms** | **p value** | **R-sq (adj):** | 0.8 |
| *s(Time)* | <2e-16*** | **Deviance explained:** | 80.7% |
| *s(Replicates)* | <2e-16*** | ***n*:** | 810 |
| **ROU Oxic Cy2 Data** | | | |
| **Parameters** | **Std. Error** | **t value** | **Pr(>\|t\|)** |
| *Fe^+3^ amendment* | 1.42 | -0.6 | 0.6 |
| *SO_4_^2-^ amendment* | 1.40 | -0.8 | 0.4 |
| *LagCO_2_* | 0.02 | 49.4 | <2e-16*** |
| **Smooth Terms** | **p value** | **R-sq (adj):** | 0.92 |
| *s(Time)* | 0.18 | **Deviance explained:** | 91.7% |
| *s(Replicates)* | 2.64e-05*** | ***n*:** | 623 |

CO_2_^(t)^: CO_2_ production at time t

﻿﻿Treatment: categorical fixed effect (Fe^3+^, SO_4_ ^2-^, Control)

﻿﻿Cycle: categorical fixed effect (e.g., Cy1, Cy2)

﻿﻿LagCO_2_^(t-1)^: previous value of CO production to model autocorrelation

﻿﻿U_Site_, U_Replicate_: random effects for site and replicate

ε: residual error

*f* (Time): smooth function of time to model temporal trends


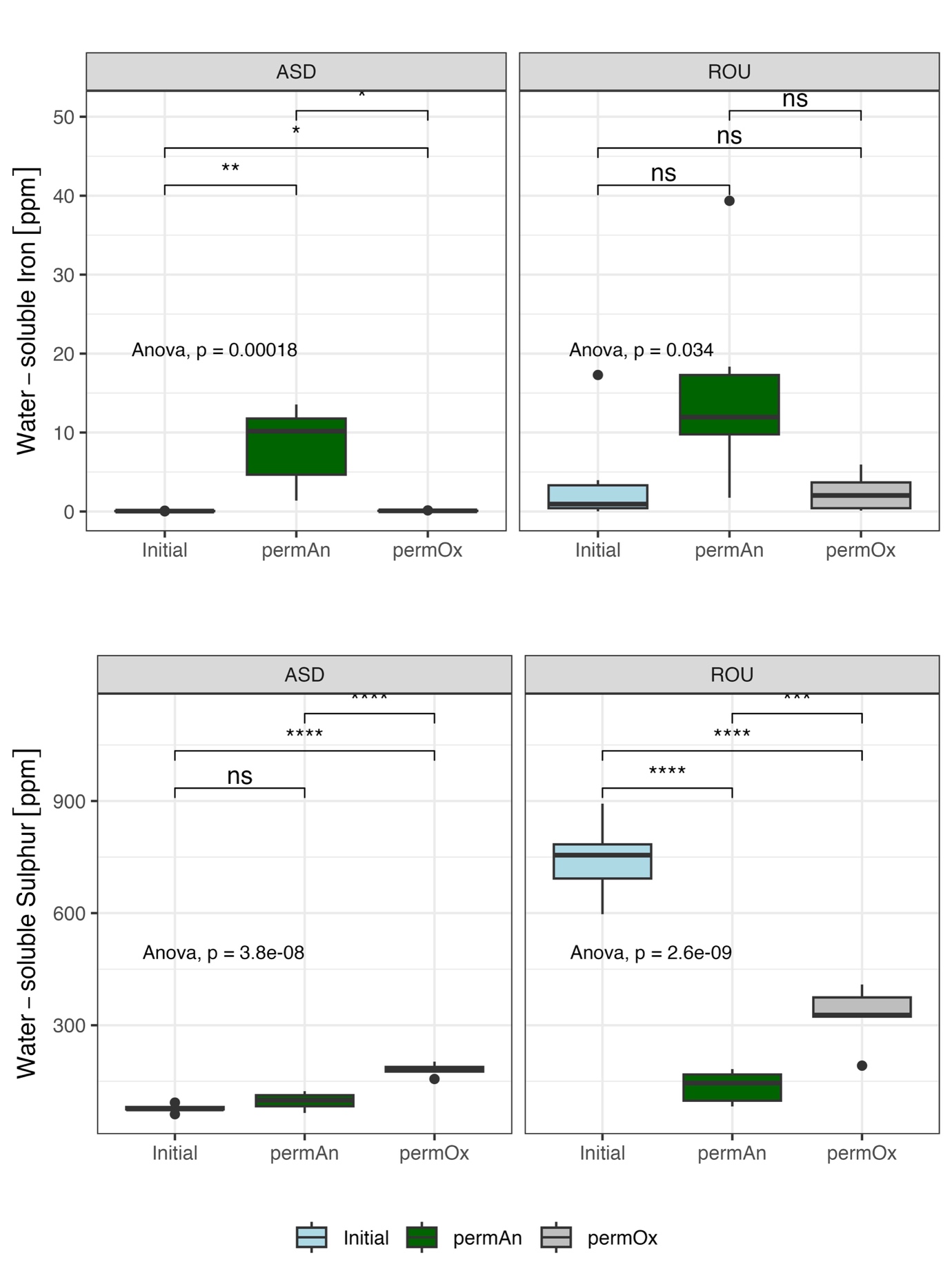


**Fig. S1.** Water-soluble iron and sulphur concentrations from two field sites—Assendelft (ASD) and Rouveen (ROU)—measured at the start of the experiment (initial field values) and after incubation under either permanently anoxic (permAn) or permanently oxic (permOx) laboratory conditions.

**Table S5.** Cumulative CO_2_ emissions [μgC-CO_2_/gC/h] with standard deviations (sd) of control and treatment samples under the oxic and anoxic conditions from Assendelft (ASD) and Rouveen (ROU) field sites.

| Site | Condition | Treatment | Cycle | Avg CO_2_cum [μgC-CO_2_/gC/h] | sd |
| --- | --- | --- | --- | --- | --- |
| ASD | Anoxic | C | Cy1 | 424.3 | 31.3 |
|  |  | Fe |  | 697.7 | 38.9 |
|  |  | S |  | 620.3 | 54.1 |
|  |  | permAn |  | 442.8 | 58.2 |
| ASD | Oxic | C |  | 815.9 | 35.5 |
|  |  | Fe |  | 701.3 | 19.5 |
|  |  | S |  | 754.9 | 73.0 |
|  |  | permOx |  | 542.2 | 29.8 |
| ASD | Anoxic | C | Cy2 | 1160.7 | 133.8 |
|  |  | Fe |  | 1574.9 | 216.4 |
|  |  | S |  | 1388.3 | 149.1 |
|  |  | permAn |  | 922.1 | 84.3 |
| ASD | Oxic | C |  | 2450.9 | 138.9 |
|  |  | Fe |  | 1678.7 | 210.1 |
|  |  | S |  | 2085.6 | 245.7 |
|  |  | permOx |  | 628.1 | 4.7 |
| ROU | Anoxic | C | Cy1 | 464.5 | 118.3 |
|  |  | Fe |  | 859.9 | 75.2 |
|  |  | S |  | 891.1 | 99.5 |
|  |  | permAn |  | 577.3 | 89.9 |
| ROU | Oxic | C |  | 2855.3 | 304.2 |
|  |  | Fe |  | 2416.1 | 128.6 |
|  |  | S |  | 2169.6 | 208.4 |
|  |  | permOx |  | 1680.5 | 152.5 |
| ROU | Anoxic | C | Cy2 | 1992.2 | 428.0 |
|  |  | Fe |  | 2790.2 | 665.9 |
|  |  | S |  | 2693.5 | 621.0 |
|  |  | permAn |  | 521.2 | 28.4 |
| ROU | Oxic | C |  | 3489.9 | 655.9 |
|  |  | Fe |  | 4298.1 | 588.0 |
|  |  | S |  | 4081.3 | 458.0 |
|  |  | permOx |  | 1819.8 | 234.7 |

**Table S6.** Changes of average cumulative rates of CO_2_ emissions in percentage increase (+) of decrease (-) from the treatments compared to controls

| **Site** | **Condition** | **Treatment** | **Cycle** | **Changes** | **Site** | **Condition** | **Treatment** | **Cycle** | **Changes** |
| --- | --- | --- | --- | --- | --- | --- | --- | --- | --- |
| **ASD** | Anoxic | Fe | Cy1 | + 64 % | **ROU** | Anoxic | Fe | Cy1 | + 85 % |
|  |  | S |  | + 46 % |  |  | S |  | + 91 % |
|  |  | permAn |  | + 4 % |  |  | permAn |  | + 24 % |
| **ASD** | Oxic | Fe |  | - 14 % | **ROU** | Oxic | Fe |  | - 15 % |
|  |  | S |  | - 7 % |  |  | S |  | - 24 % |
|  |  | permOx |  | - 33 % |  |  | permOx |  | - 41 % |
| **ASD** | Anoxic | Fe | Cy2 | + 36 % | **ROU** | Anoxic | Fe | Cy2 | + 40 % |
|  |  | S |  | + 20 % |  |  | S |  | + 35 % |
|  |  | permAn |  | - 21 % |  |  | permAn |  | - 74 % |
| **ASD** | Oxic | Fe |  | - 32 % | **ROU** | Oxic | Fe |  | + 23 % |
|  |  | S |  | - 15 % |  |  | S |  | + 17 % |
|  |  | permOx |  | - 74 % |  |  | permOx |  | - 47 % |
